# Review of cost-effectiveness analysis to study pediatric intestinal failure and transplant

**DOI:** 10.1016/j.intf.2025.100051

**Published:** 2025-03-18

**Authors:** Vikram K. Raghu, Kenneth J. Smith

**Affiliations:** University of Pittsburgh School of Medicine, Pittsburgh, PA, USA

**Keywords:** Decision analysis, Health utility, Short bowel syndrome, Teduglutide

## Abstract

Cost-effectiveness analysis is a comparative methodology used to determine how costs and benefits align for a given treatment when evaluated against one or more competing strategies. In intestinal failure, this may include direct comparisons between medical treatments vs transplant or individual care decisions. Teduglutide and antimicrobial lock therapy provide two recent examples of treatments in the United States that came under scrutiny due to cost. We demonstrate the use of cost-effectiveness analysis to critically examine these treatments in the US as an example that may be adapted to various healthcare contexts worldwide. We then discuss the required steps to employ this methodology more effectively in pediatric intestinal failure.

## 1. Introduction

The heterogeneity of pediatric intestinal failure requires families and healthcare providers to choose from a variety of treatment strategies to identify those which may most benefit each child. Parenteral nutrition, specialized diets, medications such as teduglutide, intestinal reconstructive surgeries, and intestine transplant may all play significant roles in optimizing a child’s condition. In deciding how to treat a child, we are often faced with the question of whether a treatment is *worth it*. To answer this fundamental question, the structured framework of cost-effectiveness analysis allows weighing the costs and benefits of a given treatment. In this review, we define cost-effectiveness analysis, detail its applications in pediatric intestinal failure, and list some key future directions.

## 2. What is cost-effectiveness analysis?

Cost-effectiveness analysis refers to any of several methodologies that simultaneously consider both costs and effectiveness of a given treatment compared to other possible treatments [1]. Often, a cost-effectiveness analysis is defined by what it is not. A cost-effectiveness analysis does not simply aim to identify the cheapest or most cost-saving treatment. Adopting a cost-effective strategy may incur greater cost than other potential treatments. However, the expected benefit should be worth that added cost.

The primary outcome of the simultaneous comparison of costs and benefits in such an analysis is the incremental cost-effectiveness ratio (ICER) [1]. The ICER is determined by finding the difference in cost between two interventions and dividing that difference by the difference in effectiveness between strategies. The result is a measure of cost per unit of effectiveness gained. For example, when measuring costs in dollars and benefits in terms of survival, the result may be the cost per additional year of life gained when adopting one intervention over another. When a decision-maker (e.g., patient or payor) determines how much they are willing to pay for the added benefit, they can use the ICER to determine whether a new intervention is worth the additional cost. When seeking treatments aimed at improving both quantity and quality of life, the quality-adjusted life-year (QALY) has become the gold standard effectiveness measure [1]. QALYs adjust the length of life by a factor determined by the quality of life lived during that time. For example, a year in good health at home would be worth more on the QALY scale than a year bedridden in the intensive care unit at a hospital.

**Fig. 1.**
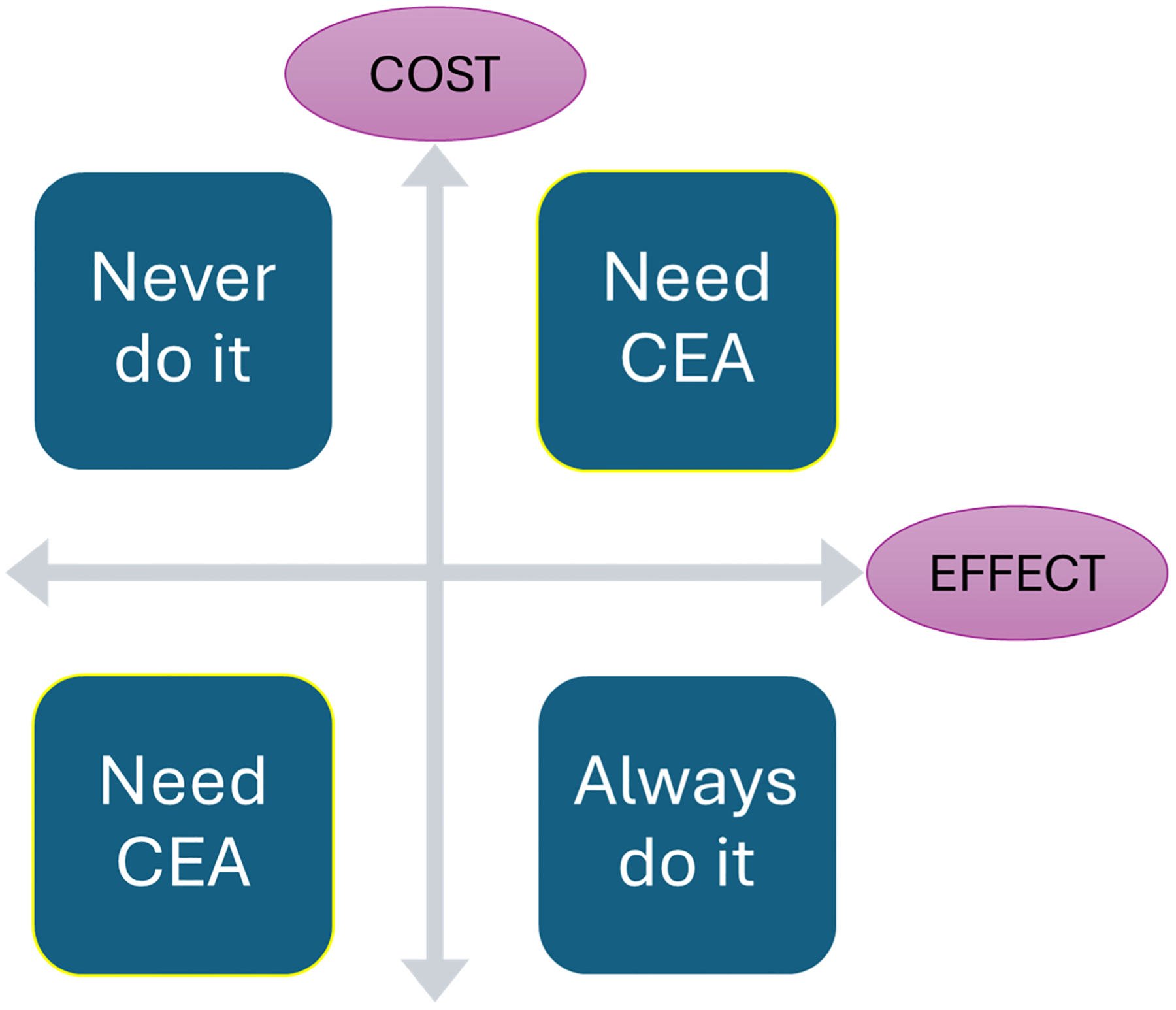
Cost-effectiveness plane. This graph demonstrates that treatments which both cost more and produce more benefit (or cost less and provide less benefit) require cost-effectiveness analysis (CEA).

When considering a new intervention, only those with a positive ICER require the framework of a cost-effectiveness analysis (Fig. 1). This is because a negative ICER implies that there is no ambiguity in the correct decision. The ICER can only be negative if a new intervention is either more expensive and less effective or if it is less expensive and more effective. In the former scenario, it would never be the correct decision to choose a treatment that is more expensive and less effective. In the latter, a less expensive and more effective treatment should always be chosen.

## 3. Why cost-effectiveness analysis in intestinal failure?

Decisions in intestinal failure are high stakes. Those with intestinal failure have high morbidity, mortality, and healthcare utilization [2–7]. However, data to support decision-making have important limitations. The gold standard for such data remains the randomized controlled trial. Yet, many barriers exist to performing such trials in intestinal failure. Intestinal failure encompasses a heterogeneous collection of disorders that may share certain features of pathophysiology and management. Specific etiologies may result in varied responses to treatments that must be considered. This may be overcome with large numbers of patients, which can be challenging in terms of the necessary financial resources and the time required. Even after overcoming time and financial barriers, some treatments may not be suitable for study in a clinical trial. Randomization involving intestine transplant would be both unfeasible and unethical. Thus, alternative frameworks are necessary to study and assist these decisions.

Cost-effectiveness analysis has been tied to the field of decision analysis, in which a simulation representing a particular disease condition may be used in place of a clinical trial [1]. These simulations can incorporate ranges of plausible costs and outcomes from multiple sources. In many cases, decision analysis can not only produce a result determining the comparative cost-effectiveness of treatments but may also identify which data have the most substantial effects on result. Even if such studies cannot replace clinical trials, they can be highly informative in focusing trial efforts on the most critically needed data.

Around the world, cost-effectiveness analysis may play an even greater role in justifying treatments for intestinal failure. Many health systems where a single national payor may be responsible for the health of the country make decisions of what treatments their constituents may receive based on the ICER. For example, the National Health Service in the United Kingdom generally requires new treatments to have an ICER compared to established interventions less than £ 20,000–30,000 per QALY gained (roughly $50,000/QALY in US dollars) be considered cost-effective enough to be implemented [8]. In many countries with smaller gross domestic products per capita, the necessary ICER for approval may need to be even lower. It is critical to remember that the budget impact of a treatment may have more to do with the prevalence of that condition than it does with how cost-effective that treatment might be. Thus, although some treatments in intestinal failure may be expensive, their overall budget impact may be limited by the rarity of their use.

Examples of cost-effectiveness studies in pediatric intestinal failure in the United States

### 3.1. Case study 1: Teduglutide

The successful launch of teduglutide revolutionized the approach to short bowel syndrome. Teduglutide is a glucagon-like peptide 2 analog that promotes intestinal adaptation by increasing villus height and crypt depth while simultaneously aiding absorption through improved blood flow and increased transit time [9]. In the United States, the single currently available teduglutide formulation has an estimated cost of around US$400,000 per year [10]. Initial data suggested that those started on teduglutide may reduce and even discontinue parenteral support altogether but may require lifelong therapy to maintain that effect.

A 2021 study examined the cost-effectiveness of four strategies a pediatric center may employ in the era of teduglutide use for pediatric short bowel syndrome [10]. They may offer or not offer teduglutide, and they may offer or not offer intestine transplant. This study focused on strategies that could not be studied through clinical trials (i.e., it would be impossible to randomize allocation of intestine transplant).

Overall, offering both teduglutide and transplant cost US$124,353/QALY gained compared to only offering transplant [10]. This does not reach the typical US-based threshold of US$100,000 QALY. However, the price may still be justified given the rarity of the condition and the potential gains from successful use. In fact, sensitivity analysis demonstrated that reducing the overall cost of the drug by just 16 % would enable it to reach that threshold [10]. This may be accomplished through dose reduction, multi-dose vials, or careful patient selection [10,11]. While scenarios existed in which use of teduglutide may or may not be considered, the model results suggested that not offering transplant nor teduglutide would never be a cost-effective option.

An additional strength of this analysis lies in its ability to provide monetary estimates for the value of gaining additional information about a parameter. Coupling this information with an estimate of prevalence of a condition can assist decision-makers in determining how much to budget for clinical studies that can ascertain that information. For teduglutide, it was determined that obtaining accurate estimates about the likelihood of achieving enteral autonomy and the mortality risk would be the most valuable. Certainty regarding the probability of achieving enteral autonomy on teduglutide was worth about US$17,000 per patient [10]. Based on current US estimates, with an incidence of short bowel syndrome of about 24.5 per million live births [12] and approximately 3.6 million live births per year [13], we can calculate an annual incidence of short bowel syndrome of about 882 children. If, based on recent registry data, half of these children are presumed to achieve enteral autonomy in the first year, we would reach an estimate of 441 new children with short bowel syndrome with intestinal failure over a year of age each year [14]. That suggests that determining the probability of achieving enteral autonomy in children receiving teduglutide would be worth about US$7.5 million annually. This can be considered the approximate value of knowing exactly who would respond to teduglutide.

### 3.2. Case study 2: antimicrobial lock therapy

For years, clinicians in the United States recommended ethanol lock therapy as the standard preventative lock for mitigating central line-associated bloodstream infections in pediatric intestinal failure [15]. However, in 2018, orphan drug designation for the injectable ethanol product led to a significant increase in the price and thus limitations on access [16]. Using similar modeling techniques as what was used to study teduglutide, a follow-up study sought to understand the cost-effectiveness of ethanol lock therapy at the new price [17]. The primary result was obvious: when the price went from the original US$16 per day to US$1000 per day, ethanol no longer was cost-effective compared to no antimicrobial lock therapy.

Sensitivity analyses provided an additional facet to these analyses. First, in determining when lock therapy would be cost-effective, a price below US$68 per day would be cost-effective and below US$63 per day would be cost saving, both well above the original price of ethanol [17]. Moreover, in certain circumstances, even much higher prices may be justified. For example, if a potential lock therapy reduced the rate of infection from every other month to no more than twice per year, it would be worth more than US$200/day [17]. As alternative lock therapies become available in the United States, these data may support efficacy-based pricing.

## 4. Future directions

As pediatric intestinal failure care continues to evolve, decision-makers will continue to face challenges. In the immediate future, novel glucagon-like peptide 2 analogues will come with varying dosing strategies, efficacies, complication rates, and costs [18,19]. These alternatives to teduglutide will need to be considered, and strategies will need to focus on how to position therapies relative to each other. Direct comparative effectiveness trials may be supplemented by cost-effectiveness analyses to provide the key data required to better inform decision-making.

To perform these analyses, cost and outcome data must be combined with appropriate data on quality of life. While more focus has been placed on quality of life in pediatric intestinal failure recently, these studies used tools that provide assessments of current health without assessing preferences. When thinking about decision-making, the goal is to compare current health and a potential different state of health. Measurements that incorporate these preferences are called health utilities and are weighted on a scale from 0 (death) to 1 (perfect health) [1]. Specific tools have been used to measure health utilities, yet these tools have not been employed in pediatric intestinal failure. The result has been trying to adapt health utilities from adults to the pediatric population. This would suggest a rather large difference between the health utility for 7 days of parenteral nutrition per week (health utility of 0.36) versus complete enteral autonomy (0.82) [20]. However, quality of life data in pediatric intestinal failure do not reflect such a large difference between those receiving parenteral nutrition and those who have achieved enteral autonomy [21]. Thus, it is critical to measure health utilities directly in children rather than relying on adult measurements.

The analyses presented represent the perspective of the United States. In many areas around the world these analyses are required by payors to make decisions about funding new treatments. In the case of intestine transplant where access to transplant may be only available in limited geographic settings, the costs to the system may grow exponentially. Developing systems to make intestine transplant accessible financially will help to ease the global burden of intestinal failure. Cost-effectiveness analyses have been used to justify intestine transplant in certain scenarios when compared directly with lifelong parenteral nutrition. It is critical to place these analyses in the context of the need for globally equitable access to treatment.

## 5. Conclusion

In summary, cost-effectiveness analysis and the accompanying simulation modeling can provide a powerful tool in the study of rare disease such as pediatric intestinal failure. These analyses have the powerful ability to compare various treatments or management strategies without a direct randomized trial, which may involve ethical limitations regarding transplant. As the field of intestinal failure continues to grow, these analyses will play a critical role in positioning the numerous therapeutic alternatives in ways that provide the best care for each child while limiting the overall burden to the healthcare system.
